# The Evolving Role of Artificial Intelligence in Medical Science: Advancing Diagnostics, Clinical Decision-Making, and Research

**DOI:** 10.7759/cureus.91514

**Published:** 2025-09-03

**Authors:** Bhavna Singla, Saleha Afridi, Sarath Vayolipoyil, Tayyab Ahmed, Sana Afzaal, Kamran Saleem, Momina T Zobia Malik, Maryam Qudsia

**Affiliations:** 1 Internal Medicine, Erie County Medical Center Hospital, Buffalo, USA; 2 Community Medicine, Fazia Medical College, Islamabad, PAK; 3 General Medicine, Scarborough General Hospital, Scarborough, GBR; 4 Cardiology, Fatima Memorial Hospital, Lahore, PAK; 5 Medicine, Social Security Hospital, Gujrat, PAK; 6 Internal Medicine, Combined Military Hospital (CMH) Lahore Medical College and Institute of Dentistry, Lahore, PAK; 7 Radiology, Shifa International Hospital Islamabad, Islamabad, PAK

**Keywords:** cdss, diagnostics, ethical challenges, healthcare management, machine learning, medical imaging

## Abstract

Artificial intelligence (AI) is transforming medical science by advancing diagnostics, clinical decision-making, research, and healthcare management. Utilizing machine learning (ML) and deep learning, AI can analyze complex datasets, identify patterns, and generate accurate predictive insights. AI-powered imaging systems and Clinical Decision Support Systems (CDSS) support early disease detection and evidence-based treatment with accuracy comparable to expert clinicians. In medical research, AI accelerates drug discovery, genomic analysis, and clinical trial optimization by mining large datasets for therapeutic targets. In healthcare management, AI enhances resource allocation, streamlines hospital operations, and improves patient interaction through virtual assistants and telemedicine. Despite these advancements, several challenges persist, including concerns about data privacy, algorithmic bias, lack of transparency in AI models, and limited physician acceptance. Ethical integration requires explainable AI, strong regulatory frameworks, and interdisciplinary collaboration. As the field evolves, AI holds great promise in precision medicine, robotic-assisted surgery, and medical education. It is also offering personalized treatment, improved surgical outcomes, and adaptive learning tools for students. Therefore, realizing this potential depends on building trust among healthcare professionals, validating AI systems, and addressing disparities in access, especially in underserved regions. AI can reshape healthcare into a more accurate, efficient, and patient-centered system worldwide with thoughtful implementation and continued innovation.

## Introduction and background

Artificial intelligence (AI) is profoundly transforming nearly every aspect of human activity and practice [1]. Improvements in diagnostics, treatment planning, research, and system management can be made in healthcare thanks to the improvements in computational power, machine learning (ML), and big data, all driving the advances in AI [2]. AI is no longer a futuristic concept, but is redefining clinical practice as practiced now, improving diagnostic accuracy, directing treatment strategies, and, ultimately, enhancing patient outcomes. However, due to the rigidity of AI in not being able to adapt to new data. In the beginning, it was applied in the form of rule-based expert systems [3]. ML and deep learning offered a solution to this in that the systems can learn from large datasets, recognize complex patterns, and give accurate diagnostic and predictive feedback [4]. Currently, AI is deployed in nearly all medical specialties, such as radiology, pathology, oncology, cardiology, neurology, mental health, as well as sleep medicine [5].

The application of AI in diagnostics is a breakthrough for AI [6]. Deep learning techniques in AI-powered imaging tools have been proven to have exceptional accuracy in identifying abnormality, surpassing that of human performance [7]. Trained on a large radiological image database, these tools can detect cancer, stroke, and lung disease with high precision, enabling earlier interventions. Imaging is just one application of AI in clinical decision-making. With AI, clinical decision support systems (CDSS) analyze electronic health records (EHRs), patient histories, and real-time data to help healthcare providers make evidence-based, informed decisions [8]. Especially useful in chronic diseases such as diabetes, cardiovascular, and cancer when timely and individual care are crucial [9].

In medical research, AI deeply accelerates drug discovery by finding promising compounds that predict outcomes and maximizing clinical trial design [10]. But by mining large biomedical datasets for insights and therapeutic targets, AI knocks down these barriers, bringing down the traditional, slow, expensive pathway for drug development. Additionally, AI significantly contributed to the fast development of COVID-19 vaccines by studying the viral structure and forecasting immune responses [11]. The scope is even larger in healthcare management, which uses AI in a significant way. From resource allocation, patient flow, to even optimizing hospital operations, predictive analytics are the way to go [12]. Patient engagement is being enhanced by AI-powered chatbots and virtual assistants, and remote monitoring systems are increasing access to care in underserved regions via telemedicine and real-time diagnostics [13].

With the integration of AI in medicine, there are challenges. Issues like data privacy and privacy need to be addressed, which calls for well-known regulatory frameworks for such systems. Many AI systems and especially deep learning models, are very opaque, so they need to be explained-solutions with explainable AI [14] are needed. Also crucial is adoption among healthcare professionals, as well as trust. As AI can improve decision-making, it is not a substitute for human practitioners’ clinical judgment and empathy. AI will be integrated effectively based on a collaborative approach in which the AI would function as a support tool [15]. This process requires ongoing education and interdisciplinary dialogue between technologists and clinicians.

AI has a great future in precision medicine, genomics, and robotic surgery. Moreover, AI could bring more intelligent, efficient, and patient-centered healthcare systems around the world. With this, personalized treatment strategies based on genetic profiles and patient histories will become ubiquitous and elevate therapeutic outcome while minimizing adverse effects [16]. Further, AI and real-time imaging can guide robotic-assisted surgeries that enhance surgical accuracy and recovery [17]. To conclude, AI has caused a medical revolution by improving diagnostics, decision-making, research, and healthcare management. Collaboration between AI experts, clinicians, and policymakers is therefore essential to ensure it continues to progress and is ethically integrated.

## Review

Materials and methods

Search strategy electronic databases, such as PubMed, Google Scholar, Xplore, and Scopus, were searched systematically. Search included peer-reviewed publications from 2014 onward on the subjects of AI in medical science, diagnosis, clinical decision making, medical research, healthcare administration, as well as medical education. The search terms used were “Artificial Intelligence in Medicine,” “Machine Learning in Healthcare,” “AI-based Clinical Decision Support Systems,” and “AI in Medical Imaging.” In order to ensure the most comprehensive coverage of relevant studies, Boolean operators (AND, OR) were used when refining the results.

Eligibility criteria

In order to ensure the selection of high-quality and relevant studies, certain eligibility criteria were set. The studies included were only studies published from 2014 onwards. They were empirical research, systematic reviews, meta-analyses, or studies about conference proceedings related to AI in medical science. Studies selected in particular are on AI-based diagnostics, clinical decision making, medical research, healthcare management, and medical education. Further, studies published in English and providing empirical data, case studies, or statistical analyses were only included. ML, deep learning, clinical decision support systems, and AI-based medical imaging were the methodologies used in these studies. Excluded were studies published before 2014, opinion pieces, non-peer-reviewed sources, and non-scientific articles. Additionally, papers offering theory about AI unrelated to medicine, loosely related to medicine, or containing no empirical data and little realization towards practical application were excluded from this study. These inclusion and exclusion criteria have been summarized in Table 1.

**Table 1:** Inclusion and Exclusion Criteria AL: artificial intelligence, ML: machine learning.

Criteria	Inclusion	Exclusion
Publication Year	Studies published from 2014 onwards	Studies published before 2014
Study Type	Empirical studies, systematic reviews, meta-analyses, and conference proceedings related to AI in medical science	Opinion pieces, non-peer-reviewed sources, and non-scientific articles
Subject	AI applications in diagnostics, clinical decision-making, medical research, healthcare management, and medical education	AI applications unrelated to medicine, general AI theory without medical relevance
Language	Studies published in English	Studies published in languages other than English
Data Availability	Studies that provide empirical data, case studies, or statistical analysis	Studies lacking data, purely theoretical discussions without practical application
Methodology	Studies using ML, deep learning, clinical decision support systems, and AI-based medical imaging	Studies without a clear methodology, opinion-based discussions, or anecdotal evidence
Outcome Measures	Impact on diagnostics, treatment planning, medical education, and healthcare management	Studies without measurable outcomes or lacking real-world applicability

Data extraction

Relevant studies were systematically screened, and data were extracted to ensure a comprehensive analysis. Study references that included authors and year of publication, as well as study design (review, review literature, empirical, systematic review, survey study) were extracted. Sample sizes were recorded where applicable to reveal the scale of the research. Furthermore, the type of AI tools used in each study was defined, including the algorithms or models used, including ML, deep learning, AI support for decision making, and robotic surgery. Studies used different data sources such as electronic medical records, imaging datasets, and literature-based data. The reported outcomes are further analyzed in the context of key medical applications: diagnostics, treatment planning, medical education, and healthcare management, in order to investigate the effect of AI in various aspects of medical science.

A qualitative and quantitative synthesis of the included studies was done. The studies were then categorized by their primary focus areas: AI in diagnostics, clinical decision making, education, and management of healthcare. We used descriptive statistics to summarize key trends such as how often AI applications are used in different medical domains and what the impact of AI on healthcare outcomes is. Recurring themes, challenges, and future directions for AI-driven medical advancements were identified by means of thematic analysis. Also, limitations and gaps in the current research were highlighted so as to guide future investigations.

Results

Study Selection Process

**Figure 1:**
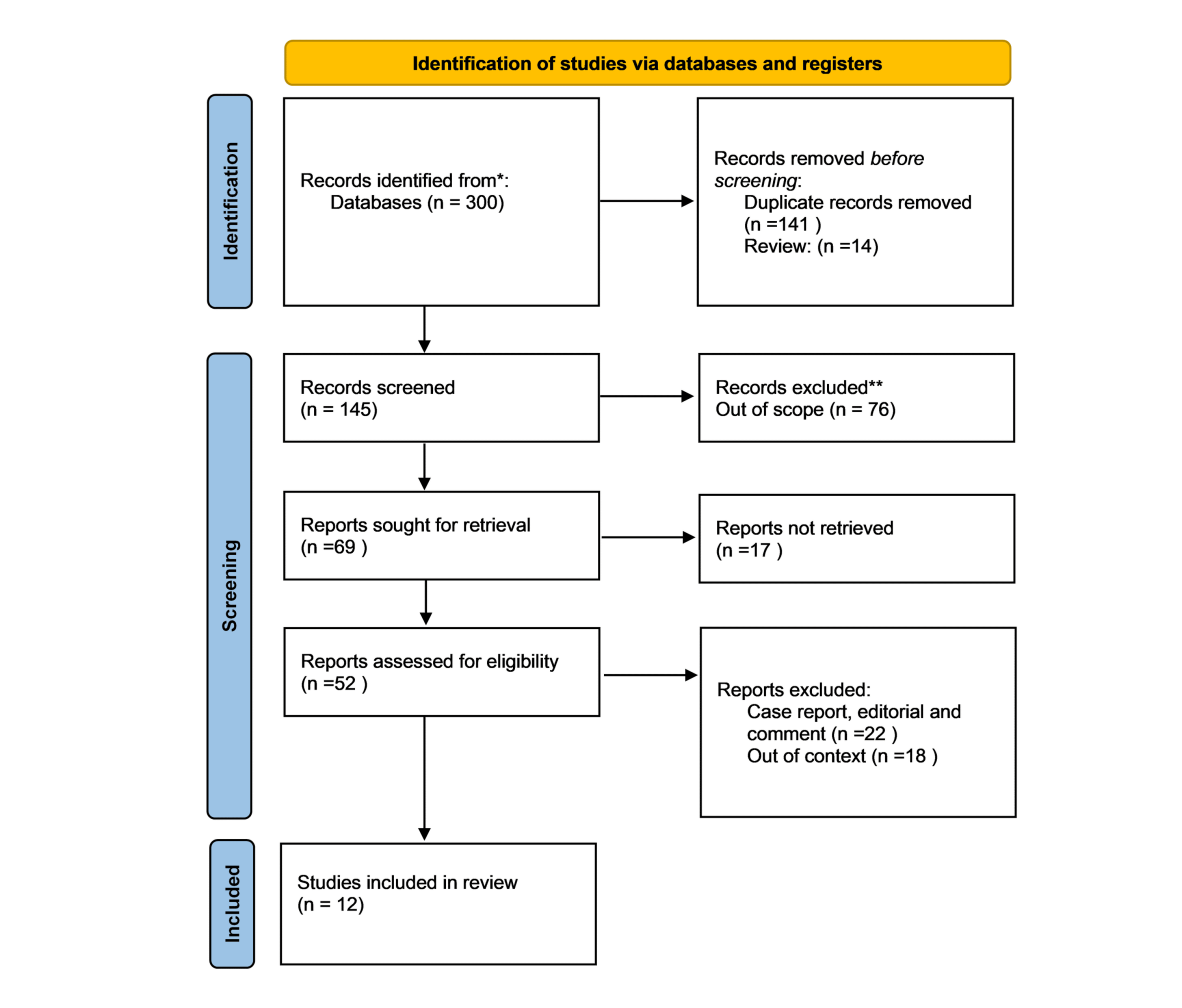
Prisma Flow Chart

A systematic study of search and selection approaches was carried out using electronic databases such as PubMed, Google Scholar, Scopus, and Xplore. Out of 300 records identified, 141 were duplicates removed. Out of an initial 145 studies, 76 were excluded from further review because they were out of scope. The 17 not retrieved were assessed for retrieval, leaving 69 reports to assess. Eligible reports from the final eligibility assessment included 22 case reports, editorials, and commentaries, and 52 other reports, of which 18 were out of context. In total, 12 studies were identified that met the inclusion criteria and were brought into this review. A flowchart illustrating the selection and evaluation process is presented in Figure 1.

We prioritized empirical studies, systematic reviews, and meta-analyses of AI applications to specific diagnostics, clinical decision making, medical research, healthcare management, and medical education. Studies published before 2014, opinion pieces, and non-peer-reviewed sources were excluded. Each of the studies demonstrated the effect of AI in medical applications, and consequently, all the displayed conclusions were data-driven and evidence-based.

Characteristics of Selected Studies

Twelve studies were selected that comprised a broad range of AI applications in medicine, from diagnostics to clinical decision making to healthcare management to medical education. Many of these studies were of a review nature, though some presented empirical analyses and a systematic review of the literature. With respect to study design, the research comprised review studies, systematic literature reviews, empirical studies, and survey-based studies. ML, deep learning, and clinical decision support systems (CDSS), AI-assisted medical imaging, and robotic-assisted surgery were employed in various forms of AI tools. Those studies used data from electronic health records (EHR), imaging datasets, clinical guidelines, and literature-based reviews. According to key findings, the applications of AI included improving diagnostic accuracy, enhancing treatment planning, optimizing healthcare delivery in rural areas, and transforming medical education through AI-based teaching models. The studies were global in geographical scope; some focused on Europe, developing countries, and the National Health Service (NHS) in the UK. But the studies also found a number of challenges: ethical issues, data privacy concerns, the transparency of AI, acceptance by physicians, and strong regulatory frameworks to guarantee safe and effective implementation.

Results show that AI is growing to be an indispensable part of medical science, providing novel solutions as well as new problems to solve in order for wider adoption and success.

The characteristics of the twelve studies included in this review are shown in Table 2. For the contents concerning the objective of this study, all the articles included in this study were of good quality. All of them went through the objective of the study thoroughly.

**Table 2:** Overview of Selected Studies on the Application of Artificial Intelligence in Medical Sciences AL: artificial intelligence, ML: machine learning.

Study Reference	Year	Location	Study Design	AI Tool Used	Type of AI	Data Source
Dilsizian et al. [18]	2014	Not specified	Review Study	IBM Watson	ML	Electronic Health Records (EHR)
Peek et al. [19]	2015	Europe	Conference Review	Not specified	ML, Data Mining	AIME Conference Papers
Hamid [20]	2016	Not specified	Review Study	Not specified	AI in Medical Imaging	Not specified
Hamet et al. [21]	2017	Not specified	Review Study	Robotic-assisted surgery, Deep Learning	Virtual and Physical AI	Not specified
Guo et al. [22]	2018	Developing Countries	Literature Review	AI-assisted Medical Techniques	AI in Rural Healthcare	Literature Data
Becker [23]	2019	Not specified	Review Study	ML Applications	AI in Risk Assessment, Disease Management	Various AI Systems in Healthcare
Petkus et al. [24]	2020	NHS (UK)	Survey Study	Clinical Decision Support Systems (CDSS)	AI in Clinical Decision Making	Specialty Societies' Responses
van Baalen et al. [25]	2021	Not specified	Theoretical Analysis	Clinical Reasoning Support Systems (CRSS)	AI in Clinical Decision Making	Not specified
Bleher et al. [26]	2022	Not specified	Theoretical Analysis	AI-driven Clinical Decision Support Systems	AI in Responsibility Attribution	Digital Tumor Board Case
Rubinger et al. [27]	2023	Not specified	Review Study	ML	AI in Research and Healthcare	Electronic Medical Records (EMR)
Kolasa et al. [28]	2024	Not specified	Systematic Literature Review	Neural Networks, SVM, Random Forest	ML in Healthcare	Various ML Studies
Zhang et al. [29]	2024	Global	Systematic Review	AI-based Teaching Models	AI in Medical Education	Web of Science, PubMed, Scopus, Xplore

Table 3 summarizes the diagnostic efficiency and clinical outcomes of twelve reviewed studies, highlighting the diverse roles of AI in medical applications. It shows how AI has enhanced diagnostic accuracy, personalized care, surgical precision, and healthcare accessibility, especially in imaging, rural health, and clinical decision-making. It also reflects emerging concerns, including ethical challenges, performance gaps, and the need for validation in AI-driven healthcare systems.

**Table 3:** Diagnostic Efficiency and Outcomes in the studies

Study Reference	Diagnostic Efficiency	Outcomes
Dilsizian at al. [18]	AI-assisted diagnostics for cardiac imaging	Enhanced personalized medicine through AI in imaging and diagnosis
Peek et al. [19]	Knowledge-based to data-driven AI trends in medicine	AI advancements in clinical decision-making and medical research
Hamid [20]	AI in medical imaging	AI improves diagnostic accuracy but raises ethical concerns
Hamet et al. [21]	AI in robotic surgery & medical decision support	Increased efficiency in robotic-assisted surgery and patient care
Guo et al. [22]	AI-enhanced healthcare for rural areas	Improved healthcare access and efficiency in developing countries
Becker et al. [23]	AI applications in risk assessment & patient care	AI improves disease risk assessment, management, and research
Petkus et al. [24]	AI in clinical decision support (CDSS)	Identified benefits and concerns of AI in physician practice
van Baalen et al. [25]	AI in clinical reasoning support	AI aids medical decision-making while maintaining physician control
Bleher et al. [26]	AI in responsibility attribution for decision support	Addressed ethical and legal concerns in AI-driven clinical decisions
Rubinger et al. [27]	AI in research and healthcare	Enhanced data processing for clinical and research applications
Kolasa et al. [28]	AI is mainly used in oncology and neurology	Reporting gaps in AI performance; need for better validation
Zhang et al. [29]	AI in medical education	AI improves teaching, evaluation, and feedback in medical training

Discussion

AI has revolutionized medical science by majorly influencing diagnostics, clinical decision making, medical research, and health care management [30]. The results of the reviewed studies demonstrate vast potential for AI in medical purposes, along with significant challenges to be dealt with to enable the secure and effective adoption of AI.

Perhaps one of the most profound implications of AI is in diagnostics, specifically, in medical imaging. With remarkable accuracy and efficiency, AI-driven tools are already helping in the detection of diseases. AI algorithms and deep learning algorithms in particular have been shown in studies to provide the same accuracy or better in analyzing radiological images as human radiologists. AI has been incorporated in diagnostic processes to provide early detection of diseases, including cancer, cardiovascular, and neurological conditions, that improve patient outcomes [31]. However, despite great improvement in diagnostic accuracy with AI, data biases, interpretability, and ethical considerations in automated diagnostics still remain worrisome.

AI-powered decision support systems (CDSS) have benefited physicians in clinical decision-making by offering evidence-based recommendations. These are systems that take advantage of massive amounts of healthcare data to learn about patient history and disease progression and to suggest the best treatment plans. Transition from decision support systems to clinical reasoning support systems (CRSS) calls for the need for AI to augment, not replace, human decision making. The studies reviewed show potential for AI to process complex data patterns more quickly than can be perceived by human clinicians, minimizing the probability of diagnostic error and developing tailored approaches to treatment. Despite this, acceptance of AI in clinical practice is still a challenge, due to physician skepticism, the requirement for transparent AI models, and regulatory concerns.

AI is no longer confined to diagnostics and decision-making; it has transformed medical research by exploiting the capacity of data to use it for generating insights and predictive modeling [32]. Genomics, drug discovery, and epidemiological researchers have made breakthroughs with the help of AI and ML algorithms. Large datasets have been analyzed by AI to identify disease risk factors and to optimize clinical trials and specifically develop targeted therapies [33]. The systematic literature review uncovered that AI has been notably impactful in oncology and neurology, where huge amounts of imaging and genomic data exist to look at. Although AI methodologies in healthcare are not yet standardized, with ongoing concerns regarding data privacy and the need for greater interpretability of AI-generated findings, researchers must address these challenges to ensure the reliability and safe integration of AI-driven medical advancements.

AI applications have also helped health care management. Hospital workflows have been optimized using AI-powered systems, administrative tasks streamlined, and patient care coordination has been improved. Predictive analytics has already been used in the healthcare industry to predict patient needs, reduce hospital readmission rates, and more efficiently allocate resources. In addition, healthcare disparities in rural and underserved areas are being addressed in part by AI. Through the application of AI-assisted medical techniques, there is a promise of bridging this healthcare gap, offering remote diagnostic support and empowering non-specialist healthcare workers with the ability to deliver quality medical services. The reviewed studies demonstrate the role AI could play in improving access to proper health care in developing countries where qualified health care providers are inadequate.

Although there are many advantages, the integration of AI into medical science is not without its difficulties. Ethical issues about the privacy of patients, data security, and the risk of algorithmic biases must be thought through to the fullest degree. Legal and moral questions about accountability in instances of medical negligence emerge due to the diffusion of responsibility in AI-driven clinical decision support systems. Reviewed studies imply that regulatory frameworks will need to adapt to address these concerns without compromising ethical and legal standards for AI applications. Moreover, there is still a need for acceptance of AI from physicians for its wide implementation. Medical professionals will have to be trained to operate alongside AI systems, and AI developers should work closely with healthcare providers to ensure a seamless integration of AI into applicable environments.

In addition, the use of AI within medical education is rapidly expanding. Real-time feedback, personalised learning paths, and more sophisticated automated assessment tools have been demonstrated by AI-based teaching models to enhance medical training. The reviewed studies suggest that AI can supplement direct teaching approaches to help students gain adaptive learning experiences based on their needs. But, to apply AI in medical education to its fullest potential, challenges lie in the disciplinary gap between AI developers and medical educators and the requirement of robust validation of AI-driven teaching methodologies.

The role of AI in medical science is rapidly evolving, with the potential to enhance diagnostic accuracy, advance data-driven clinical decision-making, accelerate medical research, and optimize healthcare management. But to truly make good on the promise of AI, it will need to be a multidisciplinary effort - one that thrives off of communication between AI experts, medical professionals, policymakers, and ethicists. Data security challenges, as well as issues of transparency, physician acceptance, and regulatory oversight, are among those that must be addressed to ensure that the AI contribution truly adds value to patient care and medical practice. With AI’s stride towards progress, ethical direction, vigorous validation processes, and a commitment to bettering healthcare for all are needed when integrating AI into medical science.

Limitations

The main limitations of this paper are the key challenges that emerge when using AI in healthcare. First, data bias, lack of standardization, and transparency of the AI-generated results are still significant concerns when AI is used (or can be used) to improve diagnostics, decision making, and research. Another limitation is AI model interpretability, in which healthcare professionals often find it difficult to understand and validate AI-generated recommendations. Moreover, ethical challenges such as patient privacy, data security, as well as fear of algorithmic biases being inherent to AI, hinder widespread AI adoption. Legal and moral questions are raised about who is accountable for medical errors in AI-assisted decision-making, when responsibility for them is diffused. In addition, the results indicate the slow introduction of AI into the medical education system due to a divide between the development and practice of the two. Lastly, although AI has the potential to improve healthcare equity, specifically for rural and underserved populations, a deficit of infrastructure and trained personnel precludes it from practical engagement. These limitations present obstacles to overcome and entail a multidisciplinary collaboration, regulatory frameworks, and continuous validation of AI technologies to assess their safe and efficient exploitation in medical science.

Recommendations

Some recommendations that follow from this research paper for effective integration of AI in the healthcare sector are offered. They should first collaborate to create transparent and explainable AI models so that physicians will trust them and patients will be safe when using them. Consistency and reliability can be improved, which requires standardization of AI methodologies, especially in diagnostics and clinical decision support systems. Furthermore, regulatory frameworks need to be updated to address ethical issues such as data privacy, security, and accountability with regard to AI-based decision-making. To ensure that AI tools are being made for practical clinical use and human oversight, it will be necessary to have collaboration between AI developers, medical professionals, and policymakers. Interdisciplinary training programs that bridge the AI technology and medical expertise gap in the adoption of AI in medical education should be accelerated. Finally, acts of expanding AI-driven healthcare solutions in the programs of rural and underserved regions can help to improve accessibility and healthcare equity, which needs to be invested in for the infrastructure, workforce training, and scalable AI applications based on the local needs.

## Conclusions

Finally, the application of AI in medical science is becoming increasingly important with its growing role in diagnostics, clinical decision-making, medical research, and healthcare governance. With AI-driven medical imaging and decision support systems, diagnostic accuracy has been increased and personalized treatment plans created. Although these benefits exist, challenges still exist in AI adoption, such as data bias, ethical issues, regulatory barriers, and physician acceptance. These challenges can only be addressed through interdisciplinary collaborations, transparent AI models, and appropriate regulatory frameworks for responsible and impactful integration of AI into healthcare. With AI evolving, its success will be determined by a blend of technology and ethics designed to optimise patient outcomes and healthcare efficiency.
